# High Below-Ground Productivity Allocation of Alpine Grasslands on the Northern Tibet

**DOI:** 10.3390/plants8120535

**Published:** 2019-11-22

**Authors:** Ben Niu, Chaoxu Zeng, Xianzhou Zhang, Yongtao He, Peili Shi, Yuan Tian, Yunfei Feng, Meng Li, Zhipeng Wang, Xiangtao Wang, Yanan Cao

**Affiliations:** 1 Key Laboratory of Ecosystem Network Observation and Modeling, Institute of Geographic Sciences and Natural Resources Research, Chinese Academy of Sciences, Beijing 100101, China; niub.12s@igsnrr.ac.cn (B.N.); zengcx.12b@igsnrr.ac.cn (C.Z.); heyt@igsnrr.ac.cn (Y.H.); shipl@igsnrr.ac.cn (P.S.); tiany.13s@igsnrr.ac.cn (Y.T.); lim.17b@igsnrr.ac.cn (M.L.); wangzp.18b@igsnrr.ac.cn (Z.W.); wangxt.16b@igsnrr.ac.cn (X.W.); caoyn.15b@igsnrr.ac.cn (Y.C.); 2 University of Chinese Academy of Sciences, Beijing 100049, China; 3 Department of Resource Management, Tangshan Normal University, Tangshan 063000, China; fengyf.13b@igsnrr.ac.cn

**Keywords:** Net primary productivity, partitioning, below ground biomass, root turnover rate, model parameters, alpine grasslands

## Abstract

The allocation of net primary production (NPP) between above- and belowground components is a key step of ecosystem material cycling and energy flows, which determines many critical parameters, e.g., the fraction of below ground NPP (BNPP) to NPP (*f_BNPP_*) and root turnover rates (RTR), in vegetation models. However, direct NPP estimation and partition are scarcely based on field measurements of biomass dynamics in the alpine grasslands on the Northern Tibetan Plateau (NTP). Consequently, these parameters are unverifiable and controversial. Here, we measured above- and belowground biomass dynamics (monthly from May to September each year from 2013 to 2015) to estimate NPP dynamics and allocations in four typical alpine grassland ecosystems, i.e., an alpine meadow, alpine meadow steppe, alpine steppe and alpine desert steppe. We found that NPP and its components, above and below ground NPP (ANPP and BNPP), increased significantly from west to east on the NTP, and ANPP was mainly affected by temperature while BNPP and NPP were mainly affected by precipitation. The bulk of BNPP was generally concentrated in the top 10 cm soil layers in all four alpine grasslands (76.1% ± 9.1%, mean ± SD). Our results showed that *f_BNPP_* was significantly different among these four alpine grasslands, with its means in alpine meadow (0.93), alpine desert steppe (0.92) being larger than that in the alpine meadow steppe (0.76) and alpine steppe (0.77). Both temperature and precipitation had significant and positive effects on the *f_BNPP_*, while their interaction effects were significantly opposite. RTR decreased with increasing precipitation, but increased with increasing temperature across this ecoregion. Our study illustrated that alpine grasslands on the NTP, especially in the alpine meadow and alpine desert steppe, partitioned an unexpected and greater NPP to below ground than most historical reports across global grasslands, indicating a more critical role of the root carbon pool in carbon cycling in alpine grasslands on the NTP.

## 1. Introduction

Net primary productivity (NPP) is a key component of global carbon cycling [1,2]. NPP partitioning of above and below ground components plays a critical role in carbon cycling of terrestrial ecosystems [3,4,5,6]. Many methods can be used for NPP estimation [7,8,9,10], of which field dynamic biomass sampling [11,12] and mathematical modeling are now widely accepted and applied to grasslands across the globe [13,14]. Generally, calibration of many ecological process models is based on field biomass sampling [1,15,16]. However, field observations at local sites are difficult to accurately scale up to larger spatial regions, and thus limiting the predictive ability of terrestrial biosphere models [15,17,18]. In addition, the temporal dynamics of NPP are full of challenges to estimate compared with the robustness of large-scale vegetation productivity patterns by one-off sampling results [19]. Thus, few reliable estimates for NPP dynamics are available, especially, because of the difficulty in measuring the below ground component of NPP in terrestrial ecosystems [16], although the carbon in alpine grasslands is stored primarily in the below-ground [20]. However, few related studies focused on below ground NPP (BNPP).

The fraction of BNPP to NPP (*f_BNPP_*), representing carbon partitioning in an ecosystem, is used as an important parameter in biosphere modeling [4]. The *f_BNPP_* of global grassland ecosystems ranges from 40% to 86% [4]. Mean *f_BNPP_* at a site is generally higher in cold and arid ecosystems meaning that warming and wetting would decrease *f_BNPP_* [2,4,11,21], while manipulation experiments have demonstrated that warming increases *f_BNPP_* in prairies [22,23]. Thus, how temperature and moisture affected the temporal response of *f_BNPP_* within sites was largely unknown because it was usually site-specific [4]. Most current reports on *f_BNPP_* estimation have focused on temperate grasslands [2,24], but few *f_BNPP_* investigations on alpine grasslands have been recorded.

Fine root production, an important component of BNPP, is estimated to comprise between 20% and 33% of global annual NPP [25,26], directly influencing carbon cycles of terrestrial ecosystems [27]. Root turnover rate (RTR), another key parameter in biosphere models [28], is the inverse of root longevity and refers to the number of times fine root biomass is replaced by each year [29]. Current conventional RTR estimation method is the ratio of BNPP to the maximum root biomass [30,31,32]. However, accurately measuring RTR is among the most problematic issues in ecosystem ecology [29,33] because RTR can be directly or interactively affected simultaneously by a large number of biotic and abiotic factors [30,34,35,36]. For example, mean annual temperature (MAT) has a positive effect on RTR, but after removing the influence of MAT, mean annual precipitation (MAP) had no effect on RTR across the globe [32]. However, the RTR spatial pattern in response to climatic gradients is not always applicable to all of the local sites for RTR prediction in response to climate change [32]. Therefore, temporal RTR estimations for different ecosystems are essential for a more reliable understanding of terrestrial carbon flux in the context of climate change.

Alpine grasslands cover an area of 1.5 million km^2^ on the Qinghai-Tibetan Plateau [37] and act as a gigantic active carbon sink [14,38], with an NPP of 177.2 Gg C year^−1^ [39]. The Northern Tibetan Plateau (NTP), where alpine grasslands account for ~37% of total grassland area on the Qinghai-Tibetan Plateau, is among the most important traditional livestock husbandry areas [40]. Previous field researches generally focused on ANPP estimations by once-off sampling of peak biomass in the middle of the plant growing season [41,42,43]. However, little is known about intra- and interannual NPP dynamics, especially BNPP. Thus, *f_BNPP_* and RTR variations of alpine grasslands are unknown on the NTP.

In this study, we measured above and below ground biomass dynamics (monthly from May to September) of four typical alpine grasslands on the NTP, i.e., an alpine meadow (AM), meadow steppe (AMS), steppe (AS), and desert steppe (ADS) from 2013 to 2015. Our objectives are to: (i) Estimate above and below ground NPP dynamics; (ii) reveal critical ecological indicators, such as *f_BNPP_* and RTR, in biosphere models; and (iii) explore climate effects on NPP dynamics and partitioning (*f_BNPP_*, RTR, and BNPP in different soil layers) on the NTP.

## 2. Materials and Methods

### 2.1. Study Sites

From west to east on the Northern Tibetan Plateau, we selected four typical alpine grassland communities, including an alpine meadow (AM) in Naqu county, an alpine meadow steppe (AMS) in Bangoin county, an alpine steppe (AS) in Nima county, and an alpine desert steppe (ADS) in Gaize county as our research sites (Table 1). Our sites were grazing exclusion using fences as the Chinese national “ecological security barrier construction program” was initiated since 2006. The mean temperatures (MAT) of these sites rang from −0.41 °C to 0.67 °C, and annual precipitation (MAP) ranges from 177 mm to 458 mm (Table 1). The climate data of these four sites, including daily temperature (T, °C), precipitation (PPT, mm), and sunshine duration (H, hours), are based on climate records from the nearest Chinese national meteorological stations.

### 2.2. Field Biomass Sampling

At each site, we conducted 14 surveys in total, including four in 2013 and five in 2014 and 2015, respectively, during the three growing seasons. We established five sampling plots (replications) along an almost 2-km-long sample line, and one sampling quadrat (0.5 m × 0.5 m in AM, and 1.0 m × 1.0 m in others) per plot. Aboveground parts of plants were clipped by species to the ground level, and all litters were collected from each quadrat. At AM, below ground biomass was sampled randomly using six soil cores with an inner diameter of 7 cm after clipping. At other sites, the soil was relatively loose and dry compared to that at AM, which caused the soil samples, especially in the deeper soil layer, to fall off easily when we used the soil auger. It was very time-consuming when we used the soil auger to sample the soil because we had to let the fallen soil samples come back. Thus, soil samples at other sites used one soil block of 0.25 m × 0.25 m. However, results from the field investigations at AM showed that the magnitudes of below-ground biomass from soil cores and soil block were comparable by a simple area conversion. Thus, file-based observations confirmed the confounding effects from different below ground sample methods in this study could be ignored. All of the below-ground biomass samplings were cut to a maximum depth of 30 cm at 10 cm intervals (i.e., 0–10 cm, 10–20 cm, and 20–30 cm).

### 2.3. Laboratory Analysis

Root samples were soaked, cleaned under running water, and residual material was removed using a 0.5 mm sieve. The live biomass and dead matters of aboveground samples were separated, and the living and dead roots were distinguished by color, resilience, and the attachment of fine roots in each quadrat [44]. All of the plant samples were oven-dried to a constant weight at 65 °C, and dry mass was measured using a scale accurate to 0.001 g.

### 2.4. Productivity Estimation and Allocation

In this study, based on our monthly dynamic sampling of above and below ground biomasses (AGB and BGB, g m^−2^), we estimated monthly ANPP and BNPP (g m^−2^ year^−1^) separately [11]:

(1) $$ANPP=SUM\;\left(positive\;increments\;in\;AGB+\;dead\;matter_{above}\right)$$

(2) $$BNPP=SUM\;\left(positive\;increments\;in\;BGB+\;dead\;matter_{below}\right)\;$$

(3) NPP=ANPP+BNPP

Here, the positive increments in AGB and BGB are the live biomass increments between two adjacent sampling durations, that is, the difference in live biomass from one sampling period to the next. Dead $matter_{\mathrm{above}}$ and $matter_{\mathrm{below}}$ refers to standing dead matter plus litter on the above ground, and dead roots collected during each sample, respectively. We assumed that simultaneous growth, death and decomposition (i.e., continuous turnover) did not occur, and NPP was never negative during a sample interval [11]. Therefore, annual cumulative NPP values were the sum of the positive NPP and its components for one specific-year. 

Then, we calculated *f_BNPP_* and RTR (year^−1^) using the following equations [4,32,45]:

(4) $$f_{BNPP}=\mathrm{BNPP}/\left(\mathrm{ANPP}+\mathrm{BNPP}\right)$$

(5) RTR=BNPP/BGB

### 2.5. Statistical Analysis

After testing for the normality (Shapiro–Wilk test) and homogeneity of variance (Bartlett test, *P* > 0.05), we employed the one-way analysis of variance (ANOVA) and Tukey’s honestly significant difference (HSD) to evaluate the NPP differences among different grassland types and sampling years. We also performed repeated measures ANOVA (linear mixed effect models) to determine the main and interactive effects of alpine grassland types and climate factors on ANPP, BNPP, NPP, *f_BNPP_*, and RTR, in which alpine grassland type was a fixed effect and climate factors were random effects. We also analyzed the responses of NPP (and its components) dynamics and allocation in response to climate variations (T, PPT, and sunshine duration) using simple linear regressions and all subsets regression (all-possible-regression procedure) based on the *Regsubsets* function in the Leaps package of R software [46,47]. Relative weights (Rw) of each of the key factors controlling on NPP allocation parameters were also quantified [48]. All of the statistical and modeling procedures were performed in the R statistical computing packages (Version 3.5.1).

## 3. Results

### 3.1. Biomass Dynamics

**Figure 1:**
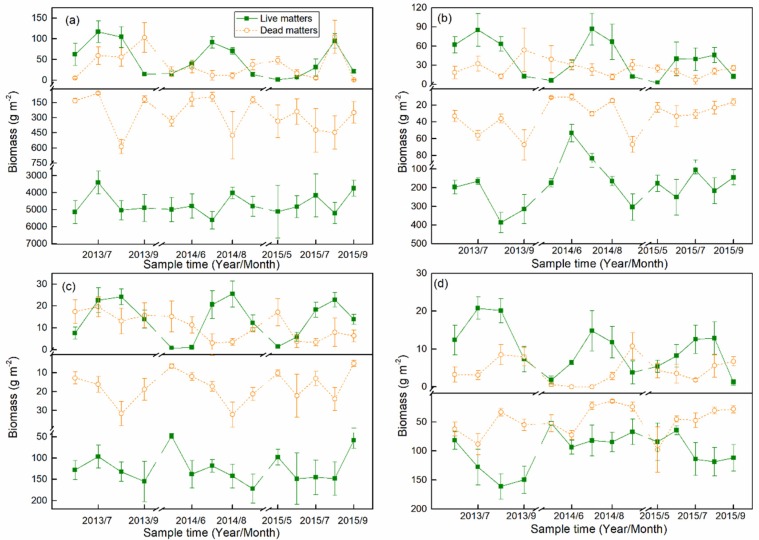
Monthly biomass dynamics for four alpine grasslands from 2013 to 2015. (**a**) Alpine meadow (AM), (**b**) alpine meadow steppe (AMS), (**c**) alpine steppe (AS), and (**d**) alpine desert steppe (ADS). “Live matters” represent the aboveground (upper insets from (**a**) to (**d**)) and belowground (bottom insets from (**a**) to (**d**)) live biomass (AGB and BGB), and the same to “Dead matters”. Error bars are the standard deviation of five repeated samples.

Seasonal dynamics of the aboveground live biomass (AGB) were generally consistent with a unimodal curve in each growing season across the four alpine grasslands, but the timing of the peak aboveground biomass varied strongly both in both time and in space (Figure 1). For example, the peak AGB of AM was in July in the first two years but was in August in 2015. Spatially, the peak AGB values were in July at AM, AMS, and ADS, but was in August at AS. However, the belowground live biomass (BGB) and the dead matter showed more complicated seasonal variations. Specifically, in AM and AMS, seasonal dynamics of BGB were similar to those of AGB (Figure 1a,b), but in AS and ADS, the seasonal patterns of AGB and BGB were completely different (Figure 1c,d). The magnitude of AGB and above ground dead matters was similar, while BGB was significantly greater than belowground dead matter (Figure 1).

### 3.2. Productivity Dynamics

**Figure 2:**
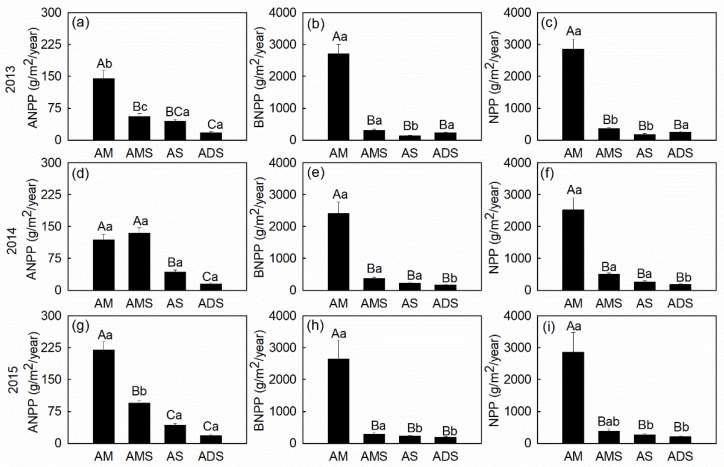
Variations in net primary production (NPP) (**c**,**f**,**i**) and its components (ANPP (**a**,**d**,**g**) and BNPP (**b**,**e**,**h**)) in four alpine grassland types on the Northern Tibetan Plateau from 2013 to 2015. Use of the same capital letter represents no significant difference among the different alpine grassland types, while the same lowercase letter represents no significant difference (*α* = 0.05) among different years. Bars indicate standard errors (n = 5).

In this study, ANPP significantly decreased along a gradient from east to west across different alpine grassland types on the NTP (Figure 2a,d,g and Table 2). Generally, the highest ANPP values were in the AM grassland, with a mean value of 160.89 g m^−2^ year^−1^ except in 2014 in AMS (Figure 2a,d,g). The ADS grassland had the lowest ANPP values, with a mean value of 16.90 g m^−2^ year^−1^. In contrast, AS and ADS showed less inter-annual ANPP variations than the other two grasslands. Overall, annual precipitation and its interaction with sunshine duration had significant effects on ANPP variations in our study (*P* < 0.05, Table 2).

**Figure 3:**
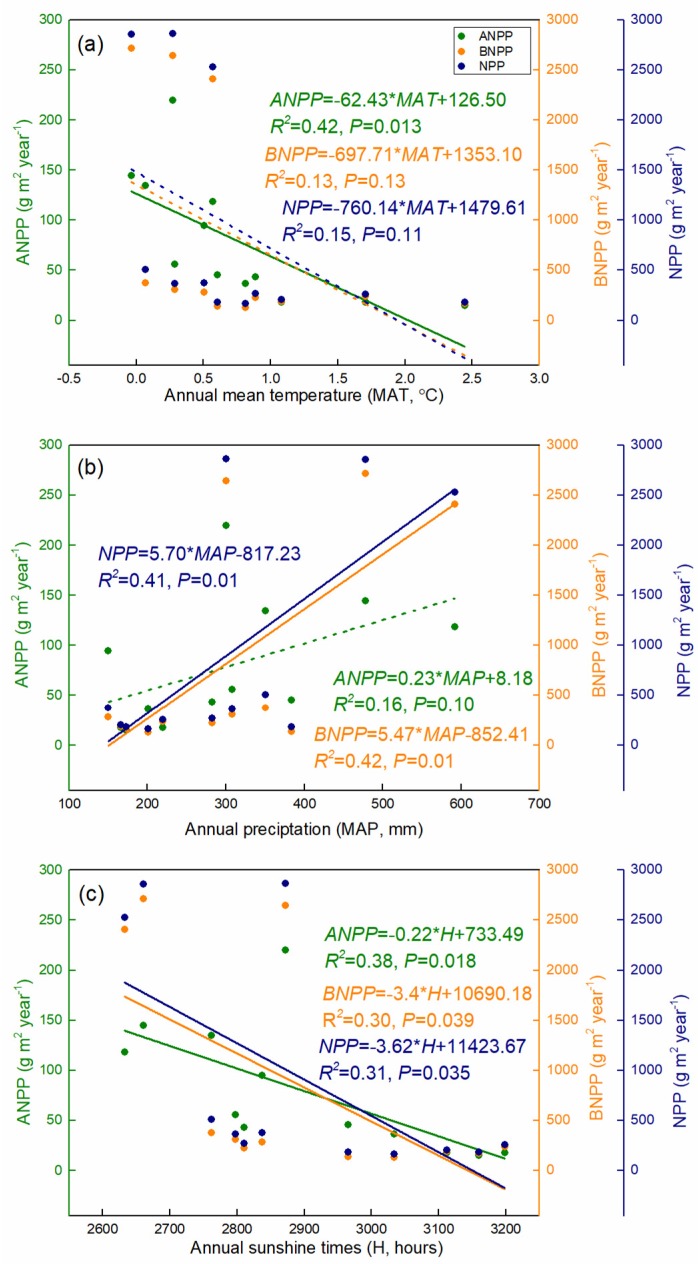
Relationships between NPP and climate variables (mean annual temperature (**a**), annual precipitation (**b**), and annual sunshine duration (**c**) in the alpine grasslands on the Northern Tibetan Plateau.

Compared with ANPP, the highest BNPP was generally present in the AM grassland with a mean values of 2588.85 g m^−2^ year^−1^ (Figure 2b,e,h). AM and AMS had no significant BNPP differences among the three growing seasons, while higher BNPP values were found in 2014 in AS and in 2013 in ADS. As the sum of ANPP and BNPP, NPP values were also the highest in AM, with a mean value of 2749.74 g m^−2^ year^−1^ (Figure 2c,f,i). The inter-annual difference of NPP in AM was negligible, while slightly higher NPP values were found in 2014 in both AMS and AS, and in 2013 in ADS (Figure 2c,f,i). Although ANPP linearly decreased with increasing annual temperature (*R*^2^ = 0.42, *P* < 0.05), neither BNPP or NPP exhibited any significant trend with temperature gradients (Figure 3a). Annual precipitation had significant and positive effects on BNPP and NPP (*R*^2^ > 0.40, *P* < 0.05), but marginal significant effects on ANPP (*R*^2^ = 0.16, *P* = 0.1) (Figure 3b). In addition, we found NPP and its components decreased significantly with increasing sunshine duration (*R*^2^ > 0. 3, *P* < 0.05) (Figure 3c). 

### 3.3. Productivity Allocation

**Figure 4:**
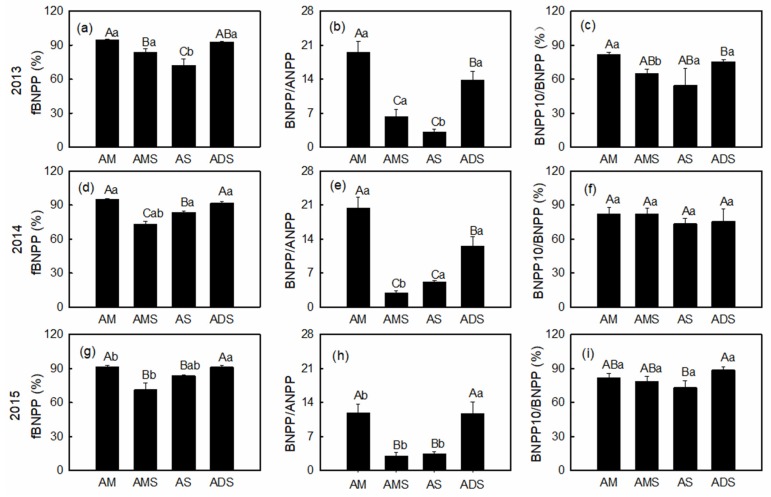
Variations in NPP partitioning parameters (*f_BNPP_* (**a**,**d**,**g**), BNPP/ANPP (**b**,**e**,**h**), and BNPP10/BNPP (**c**,**f**,**i**)) among four alpine grassland types from 2013 to 2015 on the Northern Tibetan Plateau. Use of the same capital letter represents no significant difference among different alpine grassland types, while the same lowercase letter represents no significant difference (α = 0.05) among different years. Bars indicate standard errors (n = 5).

Results showed that AM and ADS had significantly higher *f_BNPP_* values than AMS and AS (Figure 4a,d,g and Table 2). The inter-annual variation of *f_BNPP_* was non-significant in ADS, while it was significantly smaller in 2014 in the three other alpine grasslands. Similar to the *f_BNPP_* pattern, AM had the highest BNPP/ANPP value (17.26), and the inter-annual variation of BNPP/ANPP was non-significant in ADS (Figure 4b,e,h). However, BNPP/ANPP values were significantly higher in 2015 in the three other alpine grasslands (Figure 4b,e,h). In different soil layers, we found most BNPP occurred in the top 10 cm of soils, a larger BNPP10/BNPP than any other soil layers, and significantly varied among different grassland types (Figure 4c,f,i, and Table 2).

**Figure 5:**
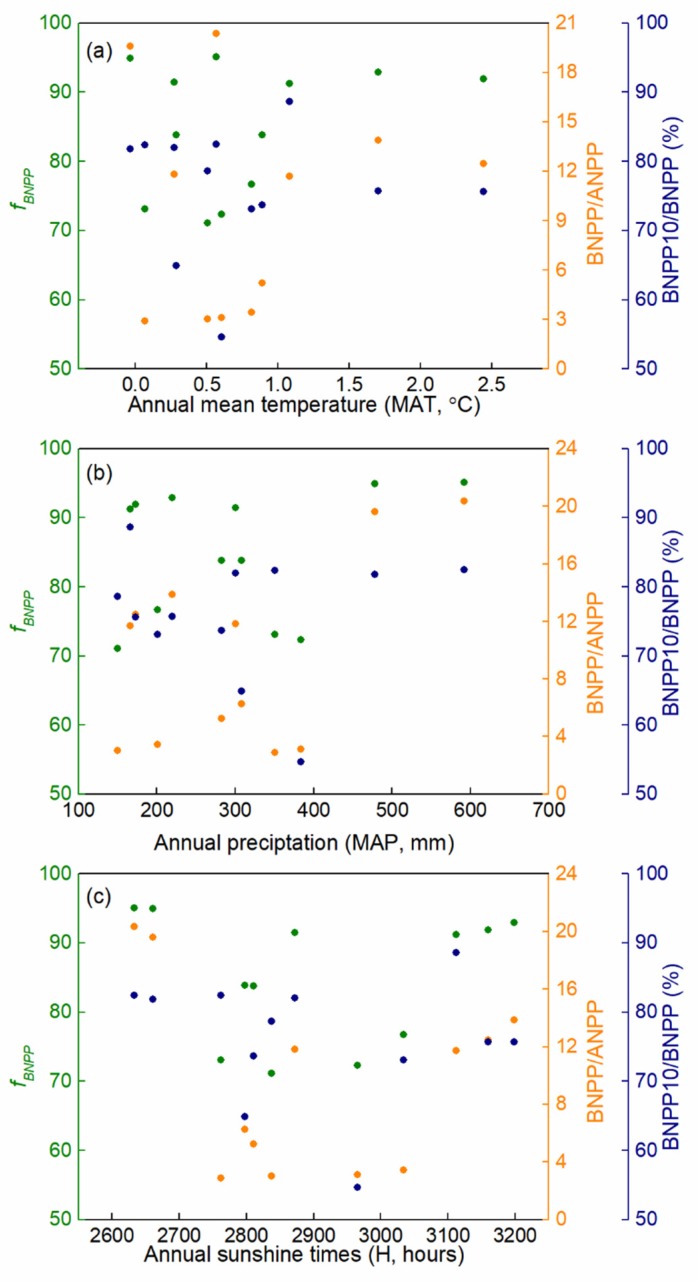
Relationships between the NPP partitioning parameters (*f_BNPP_*, BNPP/ANPP, and BNPP10/BNPP) and climate variables (mean annual temperature (**a**), annual precipitation (**b**), and annual sunshine duration (**c**) in the alpine grasslands on the Northern Tibetan Plateau.

In this study, the critical indicators of productivity allocation of alpine grasslands did not show any linear trends with climatic factors (*P* > 0.05) (Figure 5a–c). Likewise, the vertical distribution of BNPP (BNPP10/BNPP) in the soil layers also did not linearly vary with climate variables (*P* > 0.05) (Figure 5a–c). Thus, we suspected that the response of these parameters to climate factors may be non-linear. Indeed, results showed that both mean annual temperature and annual precipitation had significant and positive effects on the *f_BNPP_* and BNPP/ANPP, while BNPP10/BNPP was not significantly affected by climate factors (Table 3). However, the interaction effects between mean annual temperature and annual precipitation significantly and negatively affected *f_BNPP_* and BNPP/ANPP (Table 3).

### 3.4. Root Turnover Rates

**Figure 6:**
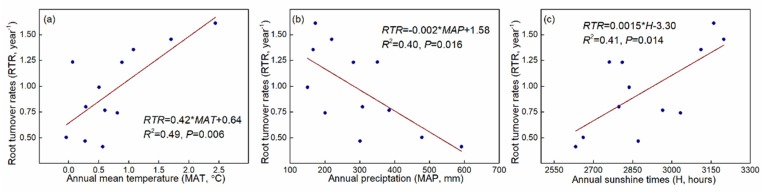
Relationships between root turnover rates (RTR) and climate variables (mean annual temperature (**a**), annual precipitation (**b**), and annual sunshine times (**c**) in alpine grasslands on the Northern Tibetan Plateau.

Root turnover rates (RTR) were generally increased in the four alpine grasslands from the alpine meadow (0.46 year^−1^) to the alpine desert steppe (1.47 year^−1^) of NTP, while there was no significant difference between the AMS and AS grasslands (Table 2 and Table 4). In contrast, only AMS and AS had significant inter-annual RTR variation, indicating a higher RTR in 2014 than in other years (Table 4). RTR exhibited a significant decrease along the increasing annual precipitation gradient of in these four alpine grasslands (*R*^2^ = 0.49, *P* < 0.01) (Figure 6a). However, both mean annual temperature (*R*^2^ = 0.40, *P* < 0.05) and annual sunshine duration (*R*^2^ = 0.41, *P* < 0.05) had significant and negative effects on RTR (Figure 6b,c). Based on comprehensive consideration of the influences of these three climate factors on RTR, we found that the mean annual temperature and annual precipitation were the key drivers of RTR variations (*R*^2^ = 0.52, *P* < 0.001), and there was no interaction between them (Table 4).

## 4. Discussion

Most previous studies on grassland NPP have focused only on the above ground component, with single-sampling biomass in each year in the AM and AS areas on the Qinghai-Tibet Plateau [19,30,49]. Few previous investigations have been concerned with ADS areas [41], and knowledge of the dynamics of NPP, especially BNPP, throughout the entire growing season is still scarce. However, many studies confirmed that the vast majority of NPP originates from BNPP in the alpine grasslands on the NTP [41,50], this implies past shortcomings are still exist for seasonal carbon sequestration assessments on the NTP. In addition, the sampling time of a single-sampling method in a year may seriously affect the results, because the timing of peak aboveground biomass strongly varied in both time and space (Figure 1). Previous study also showed that the live above ground biomass peak on the Qinghai-Tibet Plateau varied by more than forty days between 2008 and 2009 [30]. Therefore, in this study, using the monthly field measurement data of four alpine grasslands, we estimated the NPP dynamics and its components (ANPP and BNPP), which are critical for an accurate evaluation of carbon dynamics and allocation on the NTP.

### 4.1. Productivity Dynamics

Spatially, NPP showed a significant decrease along the southeast-northwest direction on the NTP, which agrees with the results derived from remote sensing images [39] and chamber-based CO_2_ flux measurements [51]. NPP estimations in this study (204.84 to 2,749.74 g m^−2^ year^−1^) were consistently within the range of global grasslands, from182 g m^−2^ yr^−1^ to 3538 g m^−2^ yr^−1^ [1]. As a part of NPP, the spatial pattern of ANPP also showed a significant decreasing trend from AM to ADS grasslands on the NTP, in accordance with the precipitation distribution [42,52]. Many studies have confirmed that ANPP in grasslands is strongly influenced by the amount and distribution of precipitation across the globe [1,2,19,53,54,55]. In this study, ANPP ranged from 94.97 g m^−2^ yr^−1^ to 160.89 g m^−2^ yr^−1^ [19], whose values were similar to the range from 83.9 g m^−2^ yr^−1^ to 125.7 g m^−2^ yr^−1^ for AM and AS grasslands located in the central-eastern region of the Qinghai-Tibet Plateau. However, our result for the AM grassland was far lower than the 579.9 g m^−2^ yr^−1^ value previously reported for the northeastern margin of the Qinghai-Tibet Plateau [30], perhaps because of site-specific dominant species and less precipitation in the alpine grasslands located in the central-eastern region of the Qinghai-Tibet Plateau.

Our study showed alpine grasslands had a relatively high BNPP, but there were no inter-annual variations across the NTP, especially in AM and AMS. We estimated BNPP for the AM grassland type to be 2588.85 g m^−2^ yr^−1^, generally higher than the previous estimate of alpine *K. humilis* meadows in Haibei, but it is still within the range of past BNPP estimates from 82.9 g m^−2^ yr^−1^ to 11183.2 g m^−2^ yr^−1^ in the similar *Kobresia*-dominated meadows on the Qinghai-Tibet Plateau (Table 5). The AS and ADS values reported here were close to those for other alpine grasslands on the plateau (Table 5). BNPP depended mainly on the dominant species and the site environment. Generally, in these cold sites on the NTP with low soil nutrient availability, plants develop a large root system in order to absorb enough water and mineral nutrients for plant growth. Thus, alpine grasslands on the infertile and dry NTP will always consist of more BNPP than ANPP[50]. For example, a ^13^C isotope labeling study conducted in an alpine meadow demonstrated a rapid and large transfer of recently assimilated carbon to the roots below ground [56].

It is well known that climate factors have significant effects on NPP variations in natural grassland ecosystems. We found a positive relationship between precipitation and NPP (ANPP and BNPP) across this region (Figure 3). Previous research also showed that the annual precipitation was the main driver that controlled the potential NPP over the recent years of the Qinghai-Tibet Plateau grasslands in recent years [14,57]. However, the temperature and sunshine duration exerted negative effects on the NPP in this study (Figure 3a,c). Generally, the increased temperature and sunshine duration should extend the growth season of plants and increase the amount of dry matter accumulation, causing a positive correlation between temperature and NPP [18]. Actually, there was a significant drop in rainfall (from 458 mm to 177 mm) as temperatures rose (from −0.41 °C to 0.67 °C) from east to west in this study (Table 1), which partly caused an apparent NPP decrease with increasing temperature. For example, a Carnegie–Ames–Stanford Approach (CASA) model-based NPP estimation in the north-Tibet also showed a negative spatial correlation between annual alpine grassland NPP and temperature (sunshine duration) [58]. In addition, warming leads to an increase in carbon sequestration as well as enhanced carbon release through ecosystem respiration, which would produce different results depending on the difference between them [59]. Besides, the decrease in root biomass density [60] would thus decrease BNPP, and the decrease in vegetation carbon density from the cold eastern to the warm western regions across our study sites [61] is also likely related to the negative temperature dependency of NPP. 

### 4.2. Productivity Allocation

All of the alpine grasslands on the NTP in this study allocated more than 75% of NPP to below ground on the NTP, which confirmed the crucial roles of roots in driving soil carbon accumulation in the alpine regions [21,70]. Compared with the results of Wu et al. 2011 [30], our results showed higher *f_BNPP_* values, especially for AM and ADS grasslands. Actually, Hui and Jackson [4] reported that 71% of the NPP of global grasslands occurs below ground. High *f_BNPP_* values have also been reported for temperate grasslands globally: 76–80% for a semi-arid steppe [24], 52–60% for a tallgrass prairie [23], and 50–67% for a grassland [31]. Overall, our results (76–93%) are higher than the global mean value and that reported for most temperate grasslands, but similar to the 77–97% range for temperate grasslands in Inner Mongolia, northern China [2] and similar alpine grasslands (70–99%) on the Qinghai-Tibet Plateau (Table 5). However, we failed to find significant and linear relationships between productivity partitioning and climate gradients [4], including precipitation, temperature, and sunshine duration, which was likely caused by smaller climate gradients in this ecoregion on the NTP. On the other hand, the inconsistent changes in rainfall and temperature across these study sites were likely to cause an apparent non-significant trend of *f_BNPP_* response to each of the climate factors due to the confounding tradeoff effects on *f_BNPP_*. For example, warming-introduced atmosphere aridity caused an increasingly intensified negative effect on alpine grassland productivity despite of the apparent positive effects from climate warming and rising CO_2_ [71]. Actually, this study illustrated a significant and opposite (negative) interaction effect between temperature and precipitation on *f_BNPP_* compared with the main positive effects of temperature and precipitation (Table 3). Therefore, direct extrapolation of *f_BNPP_* from its linear relationship with climate factors can easily lead to inaccurate results, at least for alpine grasslands on the NTP.

The ratio of BNPP/ANPP ratio in these alpine grasslands ranged from 3.92 to 17.26, i.e., higher than the global range from 0.67 to 7.3 [11]. On the basis of the functional equilibrium hypothesis, plants allocate more carbon below ground as a strategy to improve resource acquisition in dry or infertile regions and in cold regions [16]. Thus, drier, colder, and less fertile environments on the NTP result in higher BNPP/ANPP values in these alpine grasslands. Similar to *f_BNPP_*, the combined impact of temperature and precipitation significantly controlled BNPP/ANPP variation, which caused the linear response of BNPP/ANPP to climate gradients being non-significant due to the opposite change trend of climate factors from east to west in this study. 

Most BNPP was concentrated in the top 10 cm of soils (BNPP10/BNPP > 67%), and varied slightly varied among the different grassland types (76.1 ± 9.1%, mean ± SD), which was not significantly affected by climate factors (*P* > 0.1, Table 3). Previous studies also suggested that most BNPP in grasslands of grassland is found in shallower soils [31], and decreases sharply with soil depth [72], especially in alpine regions [30,50,60]. This vertical distribution of BNPP is strongly correlated with roots patterns in grassland ecosystems, as it is mainly the top soil layers that retain water and nutrients thus have higher metabolic activity. 

### 4.3. Root Turnover Rates

The RTR reflects the movement of assimilates from plant to soil, and accurate estimates of RTR could improve understanding of the vast soil carbon sink. The RTR estimated here (0.46–1.47 year^−1^) partially falls within the range (0.051–0.765 year^−1^) reported by Gill and Jackson [32] for boreal and other alpine grasslands. Along a transect from east to west at our study sites, we found RTR increased from AM (0.46 year^−1^) to ADS (1.47 year^−1^) grasslands. An increase in sunshine duration from east to west may contribute to this phenomenon (Figure 6c), as solar radiation also controls root growth and thus the input of carbon to soils in upland grasslands [73]. Soil temperature also strongly affects RTR. For example, mean annual temperature may contribute to the increasing RTR tendency based on their linear relationship [32]. Ineson, et al. [74] found that a longer duration of soil heating improved RTR by increasing root production and death in an upland grassland. Furthermore, increasing solar radiation combined with decreasing vegetation coverage and precipitation result in an increase in soil temperature, and this might also accelerate RTR along the AM to ADS transect. Warmer soil may also enhance populations of soil root-feeding herbivores [32], which also have important impacts on RTR. Thus, this study developed an optimal model (*R*^2^ = 0.52, *P* < 0.001, Table 3) that could be used to estimate RTR of alpine grasslands on the NTP.

Different RTRs also resulted from different dominant species (Table 1 and Table 5). For example, it has been reported that forest and tundra RTR variations depend on site-specific species [75,76]. Our results showed there were no significant differences in AMS and AS with respect to similar dominant species (*S. purpurea*) in this study (Table 1). In contrast, the RTR (0.46 year^−1^) in the AM (dominated by *K. pygmaea*) was similar to the value of 0.52 year^−1^ (dominated by *K. humilis*) reported by Wu et al. 2011 [30]. Besides, differences in root turnover rates were likely due to the different methods, including the root biomass sampling interval [34], and calculations of BNPP and the turnover rate [33]. For example, both the magnitude and amplitude of RTR estimations were significantly higher than the RTR estimated on the plateau by Gill’s algorithm [7] (Table 5). Thus, in this study, we employed a relatively comprehensive methods to take biomass dynamics into account in estimating BNPP, which reduced the errors of RTR estimations due to single sampling to some extent. 

### 4.4. Limitations and Applications

In this study, based on month-to-month sampling of field biomass over three consecutive growing seasons, we estimated NPP dynamics and partitioning between above and below ground components at the community level in four alpine grasslands on the NTP. However, some limitations still exist. Our results showed significantly higher NPP than that reported for other similar alpine grasslands on the Qinghai-Tibet Plateau. Although different methods may result in larger differences in NPP, the influence of some critical ecological indicators, i.e., *f_BNPP_*, BNPP/ANPP, BNPP10/BNPP, and RTR, is probably smaller when using the same method to obtain ANPP and BNPP [4]. These indicators are critical parameters in most vegetable productivity models, and they are among the largest sources of uncertainty in future projections of the carbon-climate feedbacks. Therefore, this study not only reported novel insights into how productivity dynamics and allocation respond to climate factors, but also contributed to improving vegetable productivity models by using more accurate parameters for the NTP. In addition, this study provided a simple and field-based methods to estimate productivity allocation indicators, including *f_BNPP_*, BNPP/ANPP, and BNPP10/BNPP, and RTR for the alpine grasslands using climate data on the NTP (Table 3), which could be indirectly used to estimate BNPP when the aboveground biomass and climate data are known but the belowground parts are missing or difficult to obtain.

However, apart from climate effects on NPP, which were analyzed in this study, anthropogenic activities are also critical. For example, fencing and grazing are common disturbances in Tibetan natural grasslands [49] due to fencing has been widely considered an effective measure for restoring vegetative productivity [41,49,77,78]. Our sampling completely excluded the livestock grazing effects on NPP, but could not avoid the fencing effects to some extent. For example, a synthesis study showed the above and below ground biomass increased significantly during years with fencing and achieved a steady state after 15 years in China grasslands [79]. Our investigations commenced after fencing for 4–7 years, i.e., when biomass was still rapidly increasing. Because of the absence of continuously fencing grassland biomass and grazing intensity data, the fencing effect on NPP is also difficult to quantify in the current analysis. Thus, to ascertain these effects on NPP in alpine grasslands, especially their effects on BNPP, more long-term and rigorous field investigations are needed.

## 5. Conclusions

This study provided a direct estimation of NPP from monthly dynamic field biomass sampling in four typical alpine grasslands on the NTP. We found NPP varied significantly with climate gradient in these alpine grasslands. Our results suggested that alpine grasslands allocate more NPP to below ground (>75%), the bulk of which is stable in the top 10 cm of soil (76.1 ± 9.1%, mean ± SD). Specifically, high *f_BNPP_* values were found across this ecoregion, with mean values of 93.83%, 76.02%, 77.60%, and 92.01% for AM, AMS, AS, and ADS, respectively. RTRs showed significant differences among grassland types, increasing from 0.46 year^−1^ in the AM grassland to 1.47 year^−1^ in the ADS grassland. Variation of both *f_BNPP_* and RTR variation was mainly caused by site-specific mean annual temperature and precipitation. Overall, this study illustrated inter-annual NPP dynamics and allocations, demonstrated a series of critical ecology indicators for plants growth models, evaluated the climate effects on NPP, and determined these critical indicators from field measurements, which might contribute to improving vegetable productivity models for the NTP.

## Figures and Tables

**Table 1:** Geographic, climatic *, and vegetation information for our study sites.

Types	Longitude (° E)	Latitude (° N)	Elevation (m)	MAP (mm)	MAT (°C)	Dominant Species *	Other Plant Genera
AM	92.01	31.64	4532	458.15	−0.41	*K. pygmaea*	*Potentilla*, *Saussurea*, *Gentiana*
AMS	90.31	31.39	4611	341.74	−0.13	*S. purpurea*	*Oxytropis*, *Androsace*, *Edelweiss*
AS	86.91	32.08	4624	336.00	0.47	*S. purpurea*	*Rhodiola*, *Sinapis*, *Heteropappus*
ADS	83.25	33.17	4711	177.21	0.67	*S. purpurea* *S. glareosa*	*Oxytropis*, *Sinapis*

* Climatic variables are mean annual temperature (MAT) and precipitation (MAP) for the period from 1981 to 2014. *K. S*. represent *Kobresia* and *Stipa*, respectively. AM, AMS, AS and ADS represent alpine meadow, meadow steppe, steppe and desert steppe, respectively.

**Table 2:** Repeated measures ANOVA (linear mixed effect model) results of the main effects of alpine grassland types and three climate factors (temperature, precipitation, and sunshine duration) as well as their interactions, on NPP dynamics, allocations and root turnover rates (RTR) (n = 5, α = 0.05).

		NPP Dynamics ^‡^						NPP Allocations ^‡^						RTR ^‡^	
		ANPP		BNPP		NPP		*f_BNPP_*		BNPP/ANPP		BNPP10/BNPP ^†^			
Sources ^†^	*df*	MS	*P*	MS	*P*	MS	*P*	MS	*P*	MS	*P*	MS	*P*	MS	*P*
AGT	3	61038	***	20976609	***	23037555	***	1311.9	***	654.8	***	661.7	*	2.58	***
MAT	2	1410	0.22	55504	0.63	74614	0.58	10	0.65	3.4	0.62	149.3	0.42	0.09	0.18
MAP	2	10303	**	29613	0.72	74851	0.58	41.7	0.36	129.9	**	322.1	0.24	0.005	0.76
H	2	2617	0.1	5756	0.88	611	0.96	159.2	0.08	40.6	0.09	781.2	0.07	0.45	**
MAT * MAP	4	230	0.62	81010	0.56	89880	0.55	3.6	0.79	0.5	0.85	24.3	0.75	0.05	0.32
MAT *H	4	924	0.32	96452	0.52	116265	0.50	27	0.46	10.3	0.39	0.40	0.97	0.20	0.05
MAP *H	4	7017	**	41208	0.68	82231	0.57	0	0.99	35.8	0.11	470.2	0.16	0.12	0.12

^†^ AGT, MAT, MAP, and H represent alpine grassland types, mean annual temperature, annual precipitation, and sunshine duration, respectively. BNPP10/BNPP is the proportion of how much of the BNPP is in the top 10 cm of the soil. ^‡^ Correlation is significant at * *P* < 0.05, ** *P* < 0.01, and *** *P* < 0.001 levels (two-tailed test). MS represents mean squares.

**Table 3:** Modeling parameters and relative weights (*Rw*, %) of the optimization model between productivity allocation indicators and climate factors ^†^.

	MAT	MAP	MAT *MAP	Intercept	*R* ^2^	*P*
*f_BNPP_*	20.18 *** (53.1)	0.07 *** (46.9)	−0.06 *	59.59 ***	0.27	***
BNPP/ANPP	14.30 *** (24.8)	0.06 *** (75.2)	−0.04 **	−10.84	0.41	***
BNPP10/BNPP	12.62	0.03	−0.06	70.11	<0.1	0.57
RTR	0.30 *** (56.2)	−0.001 ** (43.8)	--	1.09	0.52	***

^†^ MAT and MAP represent mean annual temperature and annual precipitation, respectively. Correlation is significant at * *P* < 0.05, ** *P* < 0.01, and *** *P* < 0.001 levels (two-tailed test). MS represents mean squares.

**Table 4:** Estimation of annual RTR (year^−1^) on the Northern Tibetan Plateau from 2013 to 2015 *.

Year	AM (year^−1^)	AMS (year^−1^)	AS (year^−1^)	ADS (year^−1^)
2013	0.51 ± 0.05 C a	0.80 ± 0.09 B b	0.77 ± 0.10 B b	1.45 ± 0.10 A a
2014	0.42 ± 0.06 C a	1.23 ± 0.01 B a	1.23 ± 0.07 B a	1.61 ± 0.09 A a
2015	0.47 ± 0.12 C a	0.99 ± 0.16 B ab	0.74 ± 0.05 BC b	1.35 ± 0.11 A a
Mean	0.46 ± 0.04 C	1.01 ± 0.07 B	0.91 ± 0.03 B	1.47 ± 0.05 A

* Sharing of the same capital letters denotes no significant difference between grassland types in the same year or when comparing with the mean values, while the sharing of the same lowercase letters denotes no significant differences between sampling years for each of the four grassland types (α = 0.05).

**Table 5:** Cross-site comparison of the allocation of net primary productivity in the alpine grasslands on the plateau *.

Site	Lon. (° E)	Lat. (° N)	Ele. (m)	MAP	MAT	Dominant Species	ANPP	BNPP	*f_BNPP_*	RTR	Reference
Haibei	101.38	37.22	3300	412.28	0.53	*K. humilis*	303.4	898.9	0.74	/	[4]
Haibei	101.38	37.22	3250	528.0	−2.0	*K. humilis*	579.9	658.8	0.53	0.52	[30]
Haibei	101.38	37.22	3200–3400	528.0	−2.95	*K. humilis*	282.6	654.0	0.70	/	[62]
Haibei	101.38	37.22	3200–3400	528.0	−2.95	*K. humilis*	286.6	1134.1	0.80	/	[63]
Haibei	101.38	37.22	3200–3400	528.0	−2.95	*K. humilis*	282.7	654.0	0.70	/	[64]
Haibei	101.38	37.22	3250	514.0	−4.11	*K. pygmaea*	368.4	5604.8	0.94	/	[65]
Haibei	101.38	37.22	3250	514.0	−4.11	*K. humilis*	418.5	2578.0	0.86	/	[65]
Haibei	101.38	37.22	3250	514.0	−4.11	*K. tibetica*	518.4	11183.2	0.96	/	[65]
Haibei	101.20–101.38	37.48–37.45	3200–3600	546.1	−1.29	*K. humilis*	309.4	1267.9	0.80	0.32	[66]
Jinqiang	103.53	37.62	2930–3200	236.0	4.31	*K. capillifolia*	373.0	5497.9	0.94	/	[67]
Maqen	100.23	34.65	3800	495.0	−2.80	*K. pygmaea*	30.1	1704.1	0.98	/	[68]
Maqen	100.23	34.65	3800	495.0	−2.81	*K. capillifolia*	26.4	2790.7	0.99	/	[68]
Tianzhou	103.53	37.67	2900–3700	236.0	4.31	*K. humilis*	373.0	5498.9	0.94	/	[69]
Tianzhou	103.53	37.67	2900–3100	236.0	4.31	*S. purpurea-K. spp*	535.8	3739.3	0.87	/	[69]
Naqu	92.01	31.64	4532	458.1	−0.41	*K. pygmaea*	160.9	2588.9	0.94	0.46	This study
Bangoin	90.31	31.39	4611	341.7	−0.13	*S. purpurea*	94.5	321.5	0.76	1.01	This study
Nima	86.91	32.08	4624	336.0	0.47	*S. purpurea*	41.7	163.1	0.77	0.91	This study
Gaize	83.25	33.17	4711	177.2	0.67	*S.purpurea-S.glareosa*	16.9	198.3	0.92	1.47	This study
Northern Tibetan meadows							73.6	683.4	0.90	*0.32*	[41]
Northern Tibetan steppes							30.2	122.4	0.80	*0.28*	[41]
Northern Tibetan desert-steppes							13.0	86.3	0.87	*0.26*	[41]
Tibetan steppes							9.8–267.4	44.6–1834.8	0.58–0.93	/	[50]
Tibetan meadows							31.8–347.5	82.9–2784.8	0.45–0.93	/	[50]

* *K*. and *S*. represent *Kobresia* and *Stipa*, respectively. MAT (°C) and MAP (mm) are mean annual temperature and annual precipitation, respectively. ANPP (g m^−2^), BNPP (g m^−2^), *f_BNPP_* and RTR (year^−1^) were estimated from the field measurement data from corresponding references, while italicized RTR values in Zeng et al., 2015 were estimated by Gill’s algorithm [7].
